# Increased Glyphosate-Induced Gene Expression in the Shikimate Pathway Is Abolished in the Presence of Aromatic Amino Acids and Mimicked by Shikimate

**DOI:** 10.3389/fpls.2020.00459

**Published:** 2020-04-29

**Authors:** Ainhoa Zulet-González, Maria Barco-Antoñanzas, Miriam Gil-Monreal, Mercedes Royuela, Ana Zabalza

**Affiliations:** Institute for Multidisciplinary Research in Applied Biology (IMAB), Universidad Pública de Navarra, Pamplona, Spain

**Keywords:** *Amaranthus palmeri*, aromatic amino acids, shikimate, quinate, chorismate, anthranilate, glyphosate

## Abstract

The herbicide glyphosate inhibits the plant enzyme 5-enolpyruvylshikimate-3-phosphate synthase (EPSPS) in the aromatic amino acid (AAA) biosynthetic pathway, also known as the shikimate pathway. *Amaranthus palmeri* is a fast-growing weed, and several populations have evolved resistance to glyphosate through increased *EPSPS* gene copy number. The main objective of this study was to elucidate the regulation of the shikimate pathway and determine whether the regulatory mechanisms of glyphosate-sensitive and glyphosate-resistant plants were different. Leaf disks of sensitive and resistant (due to *EPSPS* gene amplification) *A. palmeri* plants were incubated for 24 h with glyphosate, AAA, glyphosate + AAA, or several intermediates of the pathway: shikimate, quinate, chorismate and anthranilate. In the sensitive population, glyphosate induced shikimate accumulation and induced the gene expression of the shikimate pathway. While AAA alone did not elicit any change, AAA applied with glyphosate abolished the effects of the herbicide on gene expression. It was not possible to fully mimic the effect of glyphosate by incubation with any of the intermediates, but shikimate was the intermediate that induced the highest increase (three-fold) in the expression level of the genes of the shikimate pathway of the sensitive population. These results suggest that, in this population, the lack of end products (AAA) of the shikimate pathway and shikimate accumulation would be the signals inducing gene expression in the AAA pathway after glyphosate application. In general, the effects on gene expression detected after the application of the intermediates were more severe in the sensitive population than in the resistant population. These results suggest that when *EPSPS* is overexpressed, as in the resistant population, the regulatory mechanisms of the AAA pathway are disrupted or buffered. The mechanisms underlying this behavior remain to be elucidated.

## Introduction

Besides to their function in protein synthesis, aromatic amino acids (AAA) serve as precursors of a wide variety of natural products that play crucial roles in plant signaling, growth and development, including responses to biotic and abiotic stresses (Zeier, 2013; Häusler et al., 2014), such as a wide range of secondary metabolites (Herrmann, 1995; Less et al., 2010). These amino acids are essential compounds in the diets of humans and monogastric livestock, which are unable to synthesize them (Galili and Hofgen, 2002; Tzin and Galili, 2010).

The AAA biosynthetic pathway, also known as the shikimate pathway, is located in plastids and can be subdivided into two steps: the prechorismate pathway and the postchorismate pathway, which via two different routes can lead from chorismate to the synthesis of Phe and Tyr or Trp (Maeda and Dudareva, 2012; Tohge et al., 2013). The prechorismate pathway consists of seven enzymatic reactions that act sequentially: D-arabino-heptulosonate 7-phosphate synthase (DAHPS), dehydroquinate synthase (DHQS), 3-dehydroquinate dehydratase-shikimate dehydrogenase (DQSD), shikimate kinase (SK), 5-enolpyruvylshikimate-3-phosphate synthase (EPSPS) and chorismate synthase (CS) (Herrmann, 1995; Dev et al., 2012). Chorismate can be used as a substrate at the first step of the postchorismate pathway by two different branches. The synthesis of Trp from chorismate is performed via six enzymatic reactions, and its first step is mediated by the anthranilate synthase (AS). The other branch, leading to Tyr or Phe biosynthesis, is mediated in its first step by the chorismate mutase (CM) for prephenate biosynthesis. After prephenate, the synthesis of Tyr or Phe may occur via two alternative pathways: the arogenate pathway, where the arogenate dehydratase (ADH) is located, and the phenylpyruvate/4-hydroxyphenylpyruvate pathway (Maeda et al., 2010). The shikimate pathway also presents lateral branches synthesizing secondary metabolites, such as quinate (Herrmann, 1995; Boudet, 2012). A scheme of the shikimate pathway is shown in Supplementary Figure 1.

As sessile organisms, plants should regulate and adjust their metabolism to dynamic changes. These changes can be at the level of amino acid metabolic networks (Less and Galili, 2009), or at the level of each biosynthetic pathway. The carbon flux through the shikimate pathway is regulated at the transcriptional (Bentley and Haslam, 1990; Maeda and Dudareva, 2012), posttranscriptional and posttranslational levels. In plants, the expression of genes seems to be closely regulated, often by the same transcription factors, such as MYB, ODO1 and EOBII (Takatsuji et al., 1992; Bender and Fink, 1998; Verdonk, 2005; Spitzer-Rimon et al., 2010).

The major factors limiting the production of essential amino acids in plants are the regulatory factors that control their synthesis by feedback inhibition loops and their efficient catabolism (Galili et al., 2016). In plants, how the carbon flux into the shikimate pathway is specifically regulated is ambiguous (Maeda and Dudareva, 2012). The entrance of carbon through this pathway is mediated by the DAHPS enzyme. While it has been described that the expression of the DAHPS is regulated in response to cellular levels of AAA in microbes (Aharoni and Galili, 2011), there is limited information about its regulation in plants. Studies with transgenic plants containing bacterial feedback-insensitive DAHPS show that this enzyme is key in the shikimate pathway and secondary metabolism derived from AAA (Tzin et al., 2012; Oliva et al., 2015). The bifurcation of the pathway toward Trp and Phe/Tyr pathways is controlled by AS and CM enzymes, as both enzymes are feedback-inhibited by the AAA of their corresponding pathways (Romero et al., 1995; Bohlmann et al., 1996). Moreover, Trp also activates CM activity to redirect flux from Trp to Phe/Tyr biosynthesis (Benesova and Bode, 1992; Lopez-Nieves et al., 2017), while Tyr activates arogenate dehydratase to redirect the flux from Tyr to Phe biosynthesis (Siehl and Conn, 1988).

The EPSPS is the target of glyphosate (Steinrücken and Amrhein, 1980), the most commonly used herbicide worldwide, which makes the study of this enzyme and shikimate interesting from an agronomic perspective. EPSPS has been studied extensively in plants, although the significance of EPSPS activity in the synthesis of AAA has still not been sufficiently addressed (Aharoni and Galili, 2011).

The repeated use of glyphosate selects for the corresponding resistance in weed populations, and one of the most problematic weed species resistant to glyphosate is *Amaranthus palmeri* S. Wats (Powles and Yu, 2010). *EPSPS* gene amplification is the main mechanism conferring glyphosate resistance in this species (Gaines et al., 2010; Chandi et al., 2012; Vila-Aiub et al., 2014). When this gene is overexpressed, the EPSPS enzyme accumulates such that the recommended field dose of glyphosate is not sufficient to inhibit EPSPS activity, and consequently, the plants survive. Although glyphosate induce upregulation of the genes of the shikimate pathway in both glyphosate-sensitive and glyphosate-resistant populations (Fernández-Escalada et al., 2017, 2019), it is not clear how glyphosate affects shikimate pathway regulation.

Despite the significance of the AAA and the herbicide glyphosate accounting for the major motivation to clarify the regulation of the shikimate pathway, it has not been completely elucidated to date. The regulatory mechanisms underlying the response of the pathway and the specific role of the intermediates or final products (AAA) have not been thoroughly investigated. Moreover, the use of a *A. palmeri* population with *EPSPS* gene amplification offers the opportunity to study if the regulation of the shikimate pathway is affected by the overexpression of one of its enzymes due to extra *EPSPS* copies.

The aim of this study was to evaluate the role of AAA and its intermediates in the regulation of the shikimate pathway. This evaluation was performed by analyzing whether AAA could revert the effects of glyphosate on the pathway and if the supply of intermediates of the pathway could mimic the glyphosate effects using *A. palmeri* glyphosate-sensitive (GS) and glyphosate-resistant (GR) plants. Finally, the shikimate content, relative gene expression level and protein content of the shikimate pathway were assessed in presence of glyphosate, intermediates and final products of the AAA biosynthetic pathway in leaf disks of GS and GR *A. palmeri* plants.

## Materials and Methods

### Plant Material

*Amaranthus palmeri* GS and GR biotypes were originally collected from North Carolina (United States) (Chandi et al., 2012; Fernández-Escalada et al., 2016). The resistance mechanism of the GR biotype was described to be *EPSPS* gene amplification (Chandi et al., 2012), with 47.5 more gene copies in GR plants than in GS plants (Fernández-Escalada et al., 2016). Seeds were surface-sterilized and germinated. The seeds were then transferred to 2.7-L tanks in a phytotron and grown in aerated hydroponic culture under controlled conditions, as described previously (Fernández-Escalada et al., 2016).

Before performing the incubation, a leaf of each plant of the A. *palmeri* GR population was harvested and immediately frozen in liquid nitrogen to determine the *EPSPS* relative genomic copy number of those individuals. After evaluating the results, 30 out of 48 evaluated plants were selected to obtain a homogeneous population with a similar relative *EPSPS* genomic copy number (between 60 and 100) to perform the experiments.

### Leaf Disk Incubation

When the GS and selected GR plants were 21 days old, leaf disks were excised from the leaves using a Harris Uni-Core puncher (4-mm diameter) (Healthcore, Bucks, United States), avoiding the leaf nerves. From each plant, the two youngest fully expanded leaves were used: one leaf to determine the shikimate content and another leaf to determine transcript levels and enzyme content measurements.

The same treatments and doses were applied in both populations (Table 1). Leaf disks were incubated for 24 h under continuous light (300 μmol m^–2^ s^–1^ photosynthetic photon flux) at 24°C. Solutions were freshly prepared, and the pH was adjusted to 7.0 with NaOH in all the treatments. Technical glyphosate (glyphosate, isopropylamine salt, 61%; Dr. Ehrenstorfer GmbH, Augsburg, Germany) was used. All other reagents were purchased from Sigma–Aldrich Chemical, Co. (St. Louis, MO, United States).

**TABLE 1:** Treatments applied to glyphosate-sensitive and glyphosate-resistant *Amaranthus palmeri* leaf disks.

**Identification**	**Treatment**	**Dose**
C	Control	
G	Glyphosate	1.75 g a.e. L^–1^
S	Shikimate	20 mM
Q	Quinate	50 mM
Ch	Chorismate	1 mM
At	Anthranilate	1 mM
AAA	Aromatic amino acids	10 μM (each AAA)
AAA+G	Aromatic amino acids + Glyphosate	10 μM + 1.75 g a.e. L^–1^

**a.e.* = *acid equivalents*.*

The incubation system is shown in Supplementary Figure 2. For shikimate content determination, individual disks were placed in individual wells of a 96-well microtiter plate containing 100 μL of treatment solutions. To obtain one disk per treatment from the same plant, as many disks as treatments were obtained from the youngest leaf of the plant. For enzyme content and transcript level determinations, 25 or 45 disks were placed, respectively, in the wells of 6-well microplates containing 2.5 mL of each treatment. In each well, disks from different leaves were incubated, but the same proportion of original plants was maintained in all treatments tested. In both incubation options, each well was considered a biological replicate.

After incubation, disks incubated in 96-well plants were washed thoroughly before freezing, and the plates were placed in a freezer (−20°C). Disks incubated in 6-well microplates were removed from the incubating medium, immediately frozen in liquid nitrogen and stored at −80°C. The experiment was repeated twice.

### Analytical Determinations

#### Shikimate Determination

The concentration of shikimate in each leaf disk located in each cell was measured according to the procedure described previously (Fernández-Escalada et al., 2016). Briefly, shikimate was extracted from the frozen-thawed leaf disks by adding 25 μL of 1.25 N HCl and incubating the plates at 60°C for 15 min. The concentration of shikimate in each cell was measured spectrophotometrically after the addition of 0.25% periodic acid/0.25% metaperiodate and 0.6 M sodium hydroxide/0.22 M sodium sulfite (Cromartie and Polge, 2000).

#### EPSPS and DAHPS Immunoblotting

Protein extraction was performed using 0.1 g of ground leaf tissue in 0.2 mL of extraction buffer (100 mM MOPS, 5 mM EDTA, 1% Triton-X 100, 10% glycerin, 50 mM KCl, 1 mM benzamidine, 100 mM iodoacetamide, 5% PVP and 1 mM PMSF). Proteins were separated by 12.5% SDS-PAGE and immunoblots were produced according to standard techniques. The protein amount loaded per well for each antibody used is specified in the figure legends. EPSPS and DAHPS antibody dilutions were 1:2000 and 1:1000, respectively (Fernández-Escalada et al., 2017). Bands were identified using a BCIP/NBT kit which was Amplified alkaline phosphatase immunoblot assay kit (BIORAD 170, BIORAD Laboratories, Inc., Hercules, CA, United States). Immunoblots were scanned using a GS-800 densitometer, and protein bands were quantified using Quantity One software (BIORAD Laboratories, Inc., Hercules, CA, United States).

#### Quantitative Reverse Transcription-PCR

The relative transcript level was measured for 11 genes of the AAA biosynthesis pathway. RNA extraction and the subsequent cDNA were performed as described in Fernández-Escalada et al. (2017). As *A. palmeri* was not sequenced, primers were designed using a related sequenced species of the Amaranthaceous family and crossed with *Arabidopsis thaliana*. Most primers were used previously (Fernández-Escalada et al., 2017). The primers for the two isoenzymes of CM (plastidics 1–3 and cytosolic 2) and the two isoforms of ADH (ADHα and ADHβ) were designed in this study. The optimal annealing temperature for each primer was determined using gradient PCR. All primers and annealing temperatures are listed in Supplementary Table 1. Melting curve analysis was conducted to verify amplification of single PCR products. Gene expression was monitored in four biological replicates. The relative transcript level was calculated using the 2-ΔΔCt method (Livak and Schmittgen, 2001). Relative transcript abundance was normalized using the normalization gene β-tubulin and each population to its own control.

### Statistical Analysis

Analysis was performed using 10 biological replicates for shikimate content determination and three or four biological replicates for enzyme content or nucleic acid determination, respectively. Replicates from both experiments were used. In the study of glyphosate and AAAs, differences between treatments for each population were evaluated by one-way ANOVA with a multiple-comparison adjustment (Tukey) at *p* ≤ 0.05. In the study using the intermediates, the difference between each parameter of untreated disks and disks of each treatment was evaluated using Student’s *t*-test (*p* ≤ 0.05). Statistical analyses were performed using IBM SPSS statistics 24.0 (IBM, Corp., Armonk, NY, United States).

## Results and Discussion

### Possible Pathway Regulation by Aromatic Amino Acids. Could AAA Revert the Changes Induced in the Pathway by Glyphosate?

**FIGURE 1:**
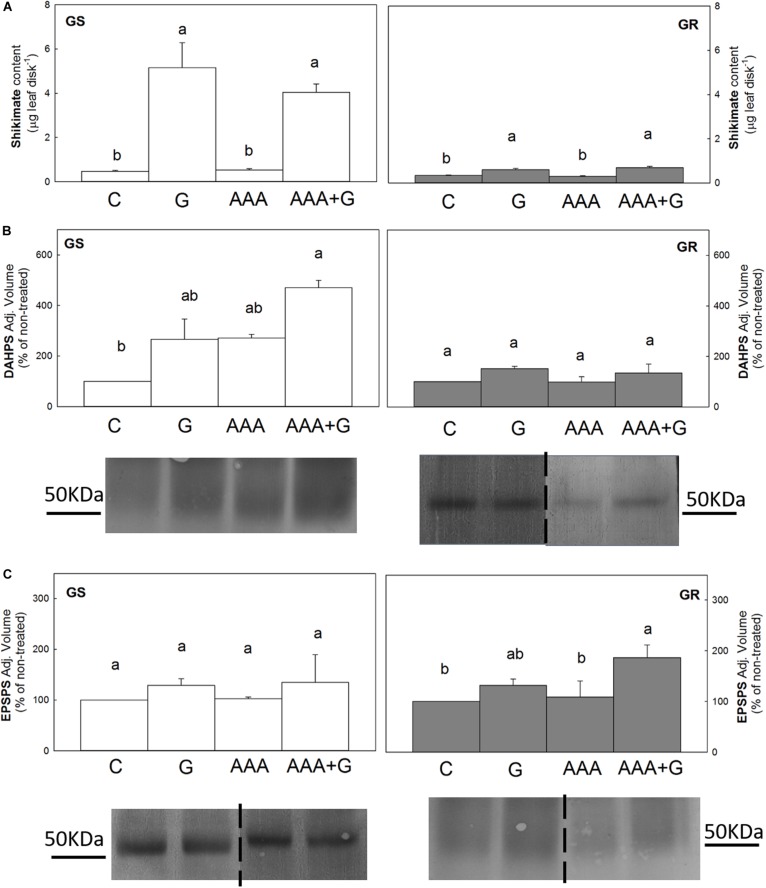
Leaf disks of glyphosate-sensitive (white bars, left; GS) and glyphosate-resistant (gray bars, right; GR) *Amaranthus palmeri* populations were incubated for 24 h with water (C), aromatic amino acids (AAA), glyphosate (G) or the combination of aromatic amino acids and glyphosate (AAA+G) (Mean ± SE). Different letters indicate differences between treatments in each population (*p*-value ≤0.05, Tukey). **(A)** Shikimate content (*n* = 10). **(B)** and **(C)** 3-deoxy-D-arabino- heptulosonate-7-phosphate-synthase (DAHPS) and 5-enolpyruvylshikimate 3-phosphate synthase (EPSPS) protein contents. Analyses of band intensity on blots are presented in graphs as the relative ratio of the control (*n* = 3). Control is arbitrarily presented as 100% of the Adjusted volume (Relative density * mm^2^). For each protein, one representative blot is shown. Original blots are shown in Supplementary Figures 3. 4. Lanes contained 40 μg of total soluble proteins for DAHPS immunoblotting and, in the case of EPSPS, 80 μg of total soluble proteins for GS and 15 μg for GR.

Shikimate content was low and similar (0.4 μg shikimate leaf disk^–1^) among untreated plants in both populations (Figure 1A). After glyphosate treatment, the shikimate content increased 11-fold in the GS population and 1.7-fold in the GR population. AAA applied alone did not modify the shikimate content in any of the populations, and when they were applied in combination with glyphosate (AAA+G treatment), the shikimate content was similar that accumulated by the herbicide-alone treatment (around 5 or 0.6 μg shikimate leaf disk^–1^ in GS and in GR, respectively).

DAHPS and EPSPS enzymes were determined by immunoblotting of (40 μg of total soluble proteins for DAHPS and, 80 μg or 15 μg for EPSPS for GS GR, respectively) (Figure 1). In relation to the untreated control, the content of these enzymes was no altered by glyphosate or AAA treatments applied alone in any of the populations. The incubation with the combination of both compounds provoked different responses depending on the population. In the GS population, the combined treatment provoked a 4.7-fold increase in DAHPS content relative to the untreated plants, but did not modify the content of EPSPS enzyme. In contrast, in the GR population, DAHPS content was not affected by this treatment, but a two-fold increase in EPSPS was observed.

**FIGURE 2:**
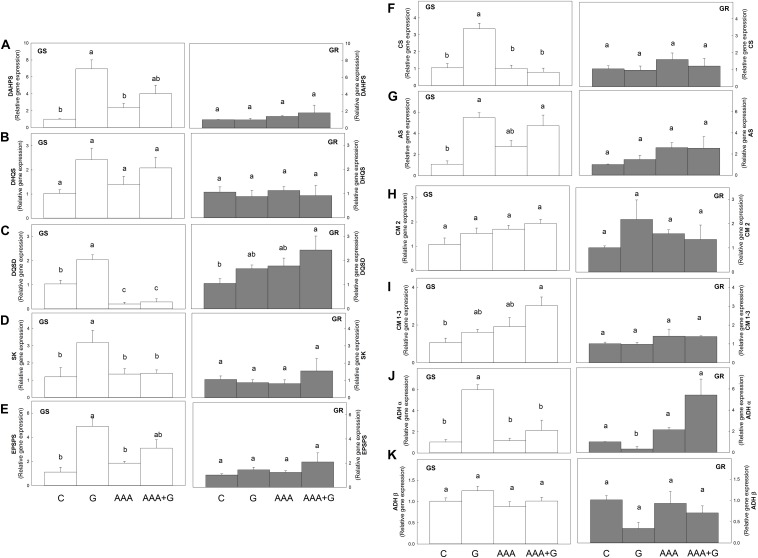
Transcript abundance of genes in the shikimate pathway was measured in *Amaranthus palmeri* leaf disks. Glyphosate-sensitive (white bars, left; GS) and glyphosate-resistant (gray bars, right; GR) populations were incubated for 24 h with water (Control, C), aromatic amino acids (AAA), glyphosate (G) or the combination of aromatic amino acids and glyphosate (AAA+G). Relative transcript abundance was normalized using the normalization gene β tubulin and each population to its own control. **(A)** 3-Deoxy-d-arabino-heptulosonate-7-phosphate synthase (*DAHPS*) **(B)** dehydroquinate synthase (*DHQS*). **(C)** 3-dehydroquinate dehydratase/shikimate dehydrogenase (*DQSD*). **(D)** Shikimate kinase (*SK*). **(E)** 5-enolpyruvylshikimate 3-phosphate synthase (*EPSPS*). **(F)** Chorismate synthase (CS). **(G)** Anthranilate synthase (*AS*). **(H)** Chorismate mutase isoform 2 (*CM2*). **(I)** Chorismate mutase isoforms 1 and 3 (*CM 1-3*). **(J)** Arogenate dehydrogenase isoform α (*ADH*α). **(K)** Arogenate dehydrogenase isoform β (*ADH*β) (Mean ± SE; *n* = 4). Different letters indicate differences between treatments in each population (*p*-value ≤0.05, Tukey).

In the GS population, glyphosate produced an increase (of more than two-fold in all cases) in the relative expression level of all the genes of the prechorismate part of the AAA biosynthetic pathway (*DAHPS, DHQS, DQSD, SK, EPSPS*, and *CS*) and only an increase of *ADH*α transcript level in the postchorismate pathway (*AS, CM2, CM1-3, ADH*α, and *ADH*β). In contrast, in the GR population, glyphosate did not induce the relative expression level of these genes in this pathway (Figure 2).

The only effect detected after AAA treatment was a five-fold decrease in the *DQSD* relative mRNA level to control plants in GS plants (Figure 2), and no effects were observed in the GR population. When AAA was applied in combination with glyphosate (AAA+G), a different response was observed in both populations. On the one hand, the GR population did not show changes in gene expression, with the exception of de *DQSD* gene that presented an increase of 2.3 times compared to the control (Figure 2). On the other hand, the presence of AAA in the combined treatment reversed the increase in transcript levels of the shikimate pathway detected after the herbicide alone in the GS population, as in this treatment, the effect of glyphosate on *DQSD, SK, CS*, or *ADH*α expression seemed to be abolished by the presence of AAA (Figure 2) and the relative mRNA level was not higher than control plants (Figure 2).

With leaf disk incubation, it was possible to reproduce the same physiological effects of glyphosate observed on whole plants. On the one hand, shikimate is a known marker of glyphosate activity (Dyer et al., 1988; Baerson et al., 2002; Zhu et al., 2008; Dillon et al., 2017), and it accumulated more in the GS than in the GR population. On the other hand, glyphosate upregulated the genes participating in the prechorismate pathway in the GS population (Figure 2), as previously described in this population (Fernández-Escalada et al., 2017) and in other species (Baerson et al., 2002; Garg et al., 2014). These results validate the incubation system used in this study to approach the regulation of the shikimate pathway by final products and intermediates.

In both populations, the accumulation of shikimate was similar in the two treatments with glyphosate, regardless of the presence of AAA (Figure 1A), suggesting that shikimate accumulation would be directly related to EPSPS inhibition and not to other physiological changes caused by the effect of the herbicide, such as a potential transitory modification of AAA content.

An increase in DAHPS or EPSPS enzyme content was not detected after incubation with glyphosate (Figure 1B,C), contrary to previous reports (Pinto et al., 1988; Gaines et al., 2010, 2011; Fernández-Escalada et al., 2017), which may be related to the short incubation period or the low dose applied. In previous experiments in which an increase in DAHPS and EPSPS content was observed, the time of treatment was 48 or 72 h (Baerson et al., 2002; Fernández-Escalada et al., 2017).

In microbes, it has been widely described that DAHPS activity is regulated in response to cellular levels of AAA (Herrmann, 1995; Tzin and Galili, 2010). However, most of the studies performed in plants suggest that DAHPS is not regulated by AAA, and only a few reports have described a regulatory effect of AAA levels on this enzyme activity *in vitro* (Graziana and Boudet, 1980; Suzich et al., 1985). Similarly, AAA treatment alone did not modify DAHPS content in this experiment (Figure 1). Indeed, the application of AAA and glyphosate together induced an increase in the DAHPS content in the GS population, supporting that in plants, the DAHPS content is not regulated by AAA levels, as it was not downregulated when AAA were externally supplied.

It has been suggested that a reduced level of AAA may act as a signal to induce the expression of the shikimate pathway and restore carbon flux through the pathway in plants (Maeda and Dudareva, 2012). In concordance with this hypothesis, a decrease in the relative expression level after the exogenous supply of AAA could have been expected. Nevertheless, this decrease did not occur, as no changes in the relative expression level were detected in the GS or GR population after AAA supply with the exception of the bifunctional enzyme *DQSD* (Figure 2). Similar to the results obtained in this study, the effect of the increase in Phe and Tyr in Arabidopsis transgenic plants on the transcriptome was low (Dubouzet et al., 2007; Tzin et al., 2009; Less et al., 2010).

The reversion of glyphosate effects on growth with exogenous AAA has been previously shown in fungi, bacteria and higher plant cell cultures (Jaworski, 1972; Gresshoff, 1979; Amrhein et al., 1983), while in higher plants, the reversion of the effect on growth effect has been detected in Arabidopsis (Gresshoff, 1979) but not in other plant species (Duke and Hoagland, 1978; Cole et al., 1980; Duke et al., 1980). Nevertheless, reversion of the physiological effects induced by glyphosate, such as anthocyanin (Hollander and Amrhein, 1980) and protein (Tymonko and Foy, 1978) synthesis, has been reported in several plant species. Similarly, in this study, supplemental AAA completely prevented one physiological effect of glyphosate: the upregulation of shikimate pathway genes.

It appears that in the GS population, the increase in gene expression of the enzymes in the shikimate pathway after glyphosate might be mediated by a transitory lack of AAA, as the exogenous supply of AAA can abolish the gene upregulation. This finding might appear contradictory to previous studies in which reduction of AAA was proposed to not elicit the increased expression of AAA pathway genes because the AAA concentrations increase with glyphosate dose (Vivancos et al., 2011; Maroli et al., 2015; Fernández-Escalada et al., 2017). In the same way, the previously reported DAHPS and EPSPS increases after glyphosate (Pinto et al., 1988; Fernández-Escalada et al., 2016) were not detected. Nevertheless, these results might be explained by the short incubation time used in this study: 24 h compared to 3 days. Indeed, previous studies have shown that in glyphosate-treated pea roots the relative content of AAA was transitorily decreased for 24 h and abolished after 3 days of treatment (Orcaray et al., 2010). The reversion effect of AAA on the upregulation of expression genes of the shikimate pathway enzymes indicated that at least in the 24 h following application of treatment, the effect was mediated by a lack of the end products of the pathway.

### Shikimate Pathway Regulation by Intermediates of the Pathway

**FIGURE 3:**
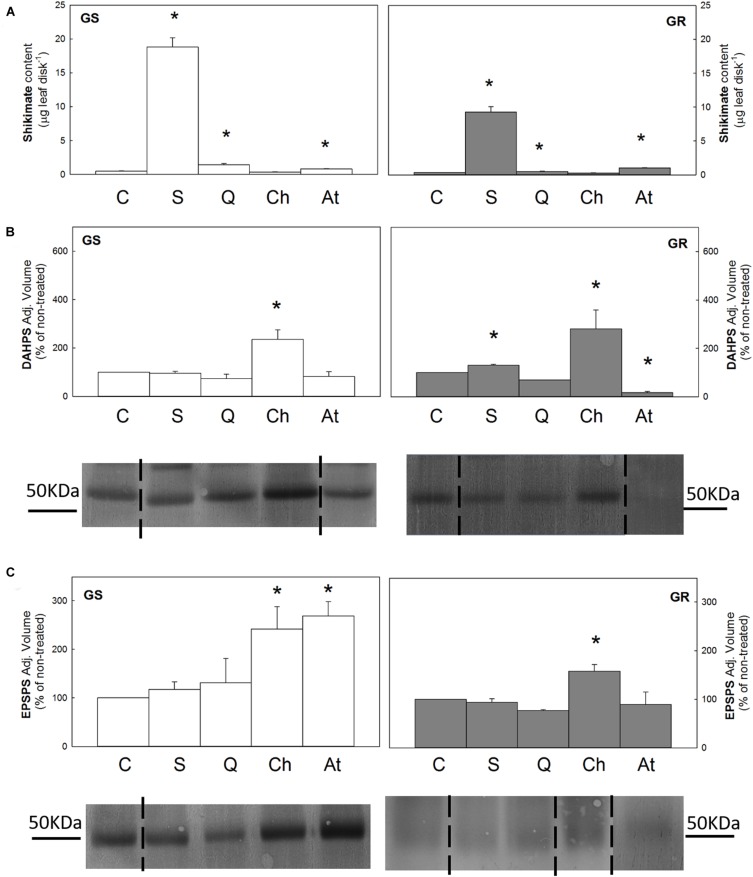
Leaf disks of glyphosate-sensitive (white bars, left; GS) and glyphosate-resistant (gray bar, right; GR) *Amaranthus palmeri* populations were incubated for 24 h with water (C), shikimate (S), quinate (Q), chorismate (Ch) or anthranilate (At) (Mean ± SE). *Symbol indicates differences between control and treatment in each population (*p*-value ≤0.05). **(A)** Shikimate content (*n* = 10). **(B)** and **(C)** 3-deoxy-D-arabino-heptulosonate-7-phosphate-synthase (DAHPS) and 5-enolpyruvylshikimate 3-phosphate synthase (EPSPS) protein contents. Analyses of band intensity on blots are presented in graphs as the relative ratio of the control (*n* = 3). Control is arbitrarily presented as 100% of the Adjusted volume (Relative density * mm^2^). For each protein, one representative blot is shown. Original blots are shown in Supplementary Figures 2, 4 Lanes contained 40 μg of total soluble proteins for DAHPS immunoblotting and, in the case of EPSPS, 80 μg of total soluble proteins for GS and 15 μg for GR.

Shikimate content was determined after incubation with the intermediates, and the pattern observed in both populations was similar (Figure 3A). Shikimate content increased in leaf disks after incubation with shikimate (40 times and 27 times in relation to the untreated control in the GS and GR populations, respectively), confirming that the compound was absorbed (Figure 3A). Shikimate content also increased after quinate treatment also (3 times in GS and 1.4 times in GR). These results showed that both in shikimate and quinate treatments, the increase observed in GS population was two times the increase observed in the GR population. Interestingly, anthranilate induced an increase in shikimate content in both populations, similar to the increase detected after quinate incubation.

DAHPS and EPSPS contents were determined after disk incubation in both populations (Figure 3). The incubation with shikimate and quinate did not induce any changes in the content of these enzymes in relation to the control disks in any population. Incubation with anthranilate elicited different responses, depending on the population and on the specific enzyme: a 2.7-fold increase in EPSPS content in GS and a 5.8-fold decrease in DAHPS expression in the GR population. Interestingly, chorismate was the only intermediate that induced a general increase in both enzymes and in both populations (DAHPS was increased 2.4 and 2.8 times and EPSPS 1.6 and 2.4 times, in GS and GR, respectively).

The incubation with shikimate induced the upregulation of more than half of the genes of the AAA pathway in the GS population (*DHQS, CS, AS, CM2, ADH*α, and *ADH*β), with the post-chorismate part being the more affected. Interestingly, this pattern was not observed in the GR population, where only *DQSD* and the *ADH*α isoform increased their relative mRNA levels after shikimate (Table 2).

**TABLE 2:** Transcript abundance of genes in the aromatic amino acid (AAA) biosynthetic pathway.

	**Glyphosate-Sensitive (GS)**			
	**Shikimate**	**Quinate**	**Chorismate**	**Anthranilate**
*DAHPS*	1.13 ± 0.02	1.57 ± 0.40	2.59 ± 0.55	0.63 ± 0.04
*DHQS*	2.46 ± 0.08*	1.92 ± 0.11*	1.91 ± 0.30*	1.23 ± 0.21
*DQSD*	1.06 ± 0.20	0.39 ± 0.04*	0.71 ± 0.08	0.87 ± 0.18
*SK*	1.85 ± 0.48	2.01 ± 0.35	1.49 ± 0.22	1.02 ± 0.18
*EPSPS*	2.51 ± 0.41	1.26 ± 0.30	0.91 ± 0.10	0.90 ± 0.18
*CS*	3.43 ± 0.74*	1.58 ± 0.14*	1.34 ± 0.10*	0.94 ± 0.12
*AS*	5.22 ± 0.11*	2.67 ± 0.22*	4.36 ± 0.79*	1.25 ± 0.16
*CM2*	2.08 ± 0.39*	3.60 ± 0.52*	1.26 ± 0.14	1.58 ± 0.31
*CM1-3*	1.62 ± 0.22	0.68 ± 0.10	0.91 ± 0.04	0.70 ± 0.05*
*ADH*α	3.56 ± 0.85*	1.68 ± 0.31	2.03 ± 0.34	0.66 ± 0.06*
*ADH*β	1.31 ± 0.06*	1.63 ± 0.17*	1.04 ± 0.04	0.93 ± 0.28

	**Glyphosate-Resistant (GR)**			
	**Shikimate**	**Quinate**	**Chorismate**	**Anthranilate**

*DAHPS*	1.06 ± 0.36	1.96 ± 0.38*	1.34 ± 0.32	0.57 ± 0.39
*DHQS*	0.53 ± 0.10	1.29 ± 0.41	0.93 ± 0.14	1.61 ± 0.18
*DQSD*	2.49 ± 0.29*	3.60 ± 0.75*	2.55 ± 0.59	1.10 ± 0.55
*SK*	0.88 ± 0.08	1.25 ± 0.32	1.14 ± 0.25	1.75 ± 0.19*
*EPSPS*	1.33 ± 0.14	2.55 ± 0.33*	1.59 ± 0.15	2.07 ± 0.52
*CS*	0.70 ± 0.17	1.51 ± 0.43	1.33 ± 0.16	0.58 ± 0.10
*AS*	0.79 ± 0.14	3.95 ± 1.16	3.82 ± 0.76	2.89 ± 0.53
*CM2*	1.49 ± 0.20	4.05 ± 1.02	1.51 ± 0.29	0.75 ± 0.24
*CM1-3*	1.00 ± 0.32	1.29 ± 0.36	0.78 ± 0.06	0.41 ± 0.06
*ADH*α	4.12 ± 0.71*	9.47 ± 1.47*	3.36 ± 0.56	2.08 ± 0.53
*ADH*β	0.93 ± 0.06	1.79 ± 0.36	0.83 ± 0.15	0.95 ± 0.11

Leaf disks of **glyphosate-sensitive** (GS) and glyphosate-**resistant** populations of *Amaranthus palmeri* were incubated for 24 h with shikimate, quinate, chorismate or anthranilate. Relative transcript abundance was normalized using the β tubulin gene and in each population to its own control. The measured genes were 3-deoxy-d-arabino-heptulosonate-7-phosphate synthase (*DAHPS*), dehydroquinate synthase (*DHQS*), 3-dehydroquinate dehydratase/shikimate dehydrogenase (*DQSD*), shikimate kinase (*SK*), 5-enolpyruvylshikimate 3-phosphate synthase (*EPSPS*), chorismate synthase (CS), anthranilate synthase (*AS*), chorismate mutase isoform 2 (*CM2*), chorismate mutase isoforms 1 and 3 (*CM1-3*), arogenate dehydrogenase isoform α (*ADH*α) and arogenate dehydrogenase isoform β (*ADH*β) (Mean ± SE; *n* = 4). *Symbol indicates differences between control and treatment in each population (*p*-value ≤0.05).

In the GS population, quinate treatment also increased the relative expression level in five out of the genes, although the level of increase was milder than that detected after shikimate, as the medium relative increase detected after shikimate was 3 times and 2.28 times after quinate (Table 2). In the GR population, four genes of the pathway (*DAHPS, DQSD, EPSPS*, and *ADH*α) were upregulated after quinate incubation. The incorporation of quinate in the shikimate pathway can occur through two different pathways: through the reversible quinate dehydrogenase to 3-dehydroquinate and through the quinate hydrolyase to shikimate (Ossipov et al., 2000). The 2.6-fold reduction in the relative gene expression in the *DQSD* gene complex in the GS population after quinate would be related to the main incorporation of quinate into the pathway after DQSD, and the increase in the flux only at that point would act as a signal to reduce the relative gene expression of the enzyme to regulate the pathway. The increase in the relative transcript level of one of the isoforms of *CM* (3.6-fold) and *ADH* (1.6-fold) genes in GS and *ADH* (9.4-fold) in GR after quinate would confirm that quinate would direct the flux to the synthesis of Tyr and Phe, as has been described recently (Zulet-González et al., 2019).

The incubation with chorismate did not alter the transcription level of *DAHPS, DQSD, SK, EPSPS, CM2, CM1-3, ADH*α, *ADH*β in any population (Table 2). In the GR population, no changes in relative gene expression were observed. In GS, only upregulation was observed in *DHQS, CS*, and *AS* genes, with the highest increase being observed for the *AS* gene (4.3-fold), the enzyme that uses chorismate as a substrate. It seems that as happens with glyphosate (Fernández-Escalada et al., 2017), the flux would be redirected toward the Trp biosynthetic pathway when chorismate is added exogenously. In addition, chorismate increased the content of the enzymes DAHPS and EPSPS (Figure 3), which does not match the results observed in the relative transcription level of the genes (Table 2), in which no changes were detected. It seems that the increase in the content of both enzymes would be due to the posttranscriptional regulation process elicited by the presence of chorismate. Nevertheless, it has to be noted that plants regulate carbon flux toward AAA biosynthesis at the transcriptional and post-transcriptional levels (Maeda and Dudareva, 2012).

Anthranilate was the intermediate that less affected the expression level of the shikimate pathway. In the GR population, only *SK* expression was 1.75-fold upregulated. In the GS population, a different response was observed on the two isoenzymes of *CM*: the plastidics 1 and 3 isoenzymes but not the cytosolic isoform 2 would be downregulated by anthranilate. In the same way, the two isoenzymes of *ADH* also showed different responses, and *ADH*α expression was the only downregulated 1.5-fold by anthranilate. In the same way, differences in the regulation of the isoforms by other metabolites of the pathway has been reported in *Beta vulgaris*, where only the activity of ADHα exhibited relaxed sensitivity to Tyr and ADHβ was strongly inhibited (Lopez-Nieves et al., 2017).

AAA biosynthesis is subjected to complex posttranscriptional and allosteric regulations (Mir et al., 2015). How carbon flow into the shikimate pathway is regulated in plants and the specific role of each intermediate remain ambiguous Siehl (1997) suggested that the inhibition of DAHPS activity by arogenate, a metabolite of the post-chorismate part of the pathway, was the key regulatory process in the shikimate pathway. In the case of glyphosate exposure, this regulatory pathway cannot occur, since chorismate and all its byproducts are not synthesized, resulting in an increase in the flux through this pathway and the accumulation of sikimate-3-phosphate (Gomes et al., 2014) and shikimate. This hypothesis was not confirmed in this study as, contrary to expectations, chorismate incubation increased the content of DAHPS and EPSPS in both populations.

The results showed that in sensitive plants glyphosate affects the shikimate pathway, and no such changes were observed in the *EPSPS* overexpressing population, consistent with its resistance to glyphosate, as has been reported before in susceptible and resistant soybeans (Marchiosi et al., 2009). Beside this, less effect on the pathway was detected on the GR than on the GS population after of the shikimate pathway intermediaries. Indeed, each intermediate induced higher changes in gene expression in the GS population than in the GR population, suggesting that the overexpression of *EPSPS* would have an effect in the regulation of the pathway, buffering or attenuating the transcriptional changes induced by the intermediates.

## Conclusion

The exogenous supply of AAA did not induce any notable change in the transcriptome of the shikimate pathway. Nevertheless, when applied in combination with glyphosate, the upregulation of gene expression detected after glyphosate was abolished, suggesting that the effect of glyphosate on relative expression level of pre-chorismate genes is mediated by a transitory lack of the final products, as detected in the GS population. Shikimate accumulation was a dose-response direct effect of EPSPS inhibition detected in both populations and cannot be abolished by increased AAA availability.

In conclusion, this study suggests that any perturbation in the shikimate pathway would provoke changes in the relative transcript level of the genes and confirm a complex regulation of this pathway with mechanisms interacting at different levels and behaving differently in each population. No intermediate fully mimicked the effect of the herbicide. Nevertheless, although the toxic effect of the herbicide could be due to a combination of different factors, shikimate incubation, similar to glyphosate, elicited upregulation of most of the shikimate pathway genes.

The effects detected after the application of the final products or the intermediates of the shikimate pathway were more severe in the GS than in the GR population, suggesting that the regulatory mechanisms that operate in the GS population are disrupted or buffered when *EPSPS* is overexpressed.

## Data Availability Statement

The datasets generated for this study are available on request to the corresponding author.

## Author Contributions

MR and AZ conceived and designed the research. AZ-G, MB-A, and MG-M conducted the research. AZ-G and MG-M analyzed the data. AZ-G and AZ wrote the manuscript. All authors have read and approved the manuscript. All authors were involved in the implementation of the experimental design and have read and approved the manuscript.

## Conflict of Interest

The authors declare that the research was conducted in the absence of any commercial or financial relationships that could be construed as a potential conflict of interest.
