# Supplementary material for: Increased Glyphosate-Induced Gene Expression in the Shikimate Pathway Is Abolished in the Presence of Aromatic Amino Acids and Mimicked by Shikimate
Source: Front Plant Sci. 2020 Apr 29;11:459. doi: 10.3389/fpls.2020.00459 (PMC7202288; doi:10.3389/fpls.2020.00459)
Supplement: Supplementary file 1 [file Data_Sheet_1.docx]

Supplementary Material

Glyphosate-induced increase in gene expression in the shikimate pathway is abolished in the presence of aromatic amino acids and mimicked by shikimate

Ainhoa Zulet-González^1^, Maria Barco-Antoñanzas^1^, Miriam Gil-Monreal^1^, Mercedes Royuela^1^, Ana Zabalza^1,*^

^1^ Institute for Multidisciplinary Research in Applied Biology (IMAB), Universidad Pública de Navarra, Campus Arrosadia s/n, 31006, Pamplona, Spain

*** Correspondence:**Ana Zabalza
ana.zabalza@unavarra.es


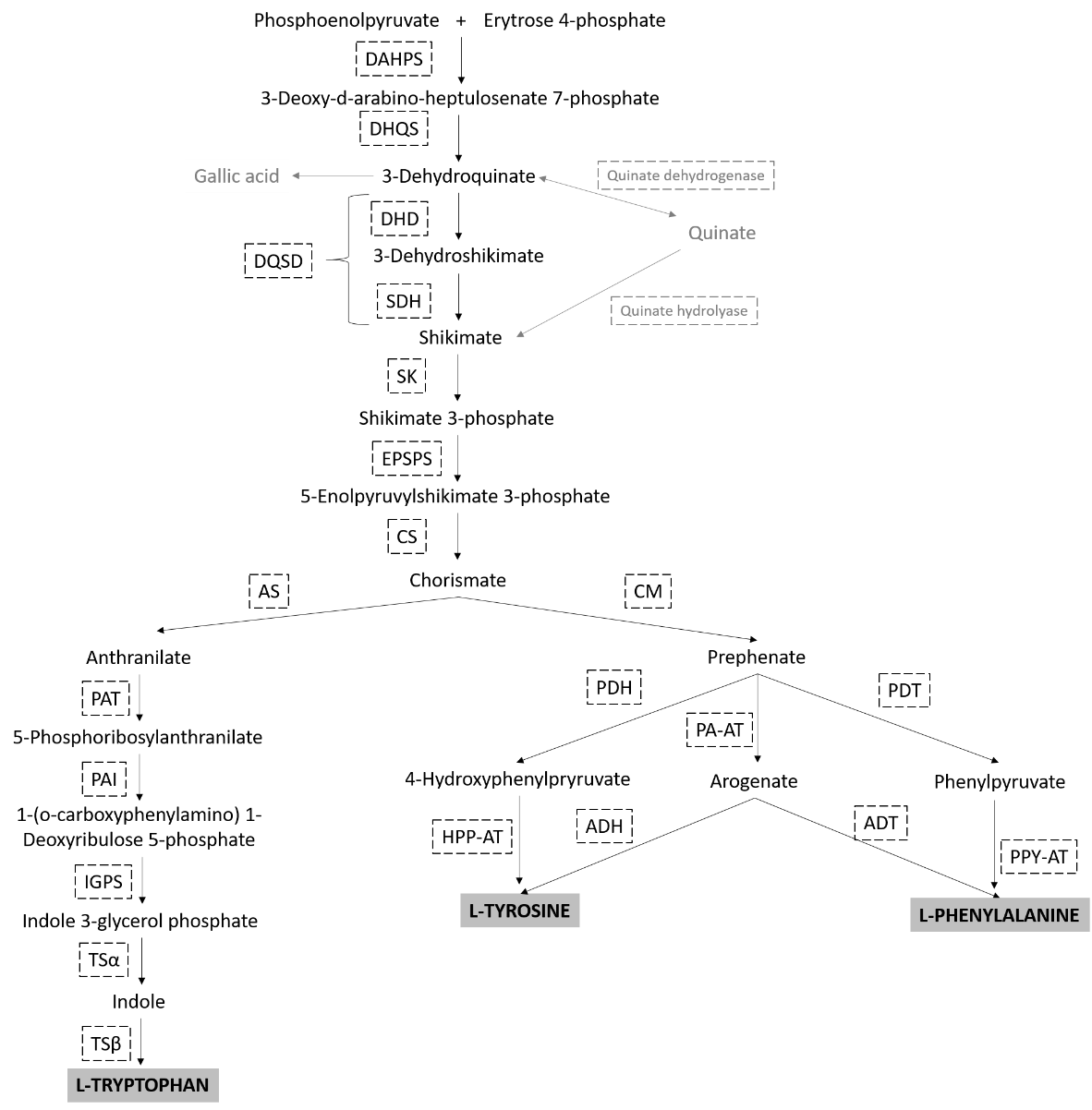


**Supplementary Figure 1**. Aromatic amino acids biosynthetic pathway in plants. The enzymes belonging the pre-chorismate pathway: D-arabino-heptulosonate 7-phosphate synthase (DAHPS), dehydroquinate synthase (DHQS), 3-dehydroquinate dehydratase (DHD), shikimate dehydrogenase (SDH), the bifunctional DHD-SDH dimer (DQSD), shikimate kinase (SK), 5-enolpyruvylshikimate 3-phosphate synthase (EPSPS) and chorismate synthase (CS). The enzymes belonging the post-chorismate pathway, towards the tryptophan synthesis: Anthranilate synthase (AS), phosphoribosylanthranilate transferase (PAT), phosphoribosylanthranilate isomerase (PAI), indole-3-glycerol phosphate synthase (IGPS), tryptophan synthase α subunit (TSα), tryptophan synthase β subunit (TSβ). The enzymes belonging the post-chorismate pathway towards the tyrosine and phenylalanine synthesis: chorismate mutase (CM), prephenate dehydrogenase (PDH), 4-hydroxyphenylpyruvate aminotransferase (HPP-AT), prephenate aminotransferase (PA-AT), arogenate dehydrogenase (ADH), arogenate dehydratase (ADT), prephenate dehydratase (PDT), phenylpyruvate aminotransferase (PPY-AT). Secondary metabolites are represented in gray and final products AAA are represented in bold capital letters and gray squared.

**B**

**A**


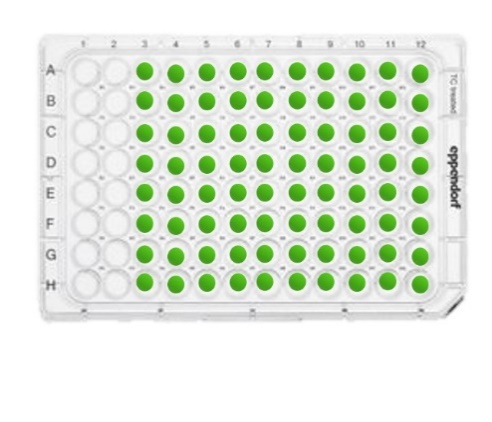

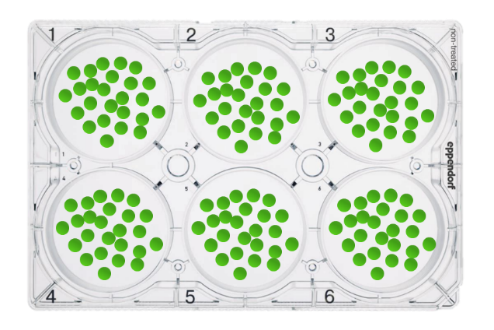


**Supplementary Figure 2.** Leaf disk incubation system. Leaf disks were excised from glyphosate- sensitive and glyphosate-resistant plants of *Amaranthus palmeri* and incubated for 24 h. One disk per well was incubated for shikimate content determination (A) and 25 or 45 disks were incubated for enzyme content and transcript level determination, respectively (B).

**
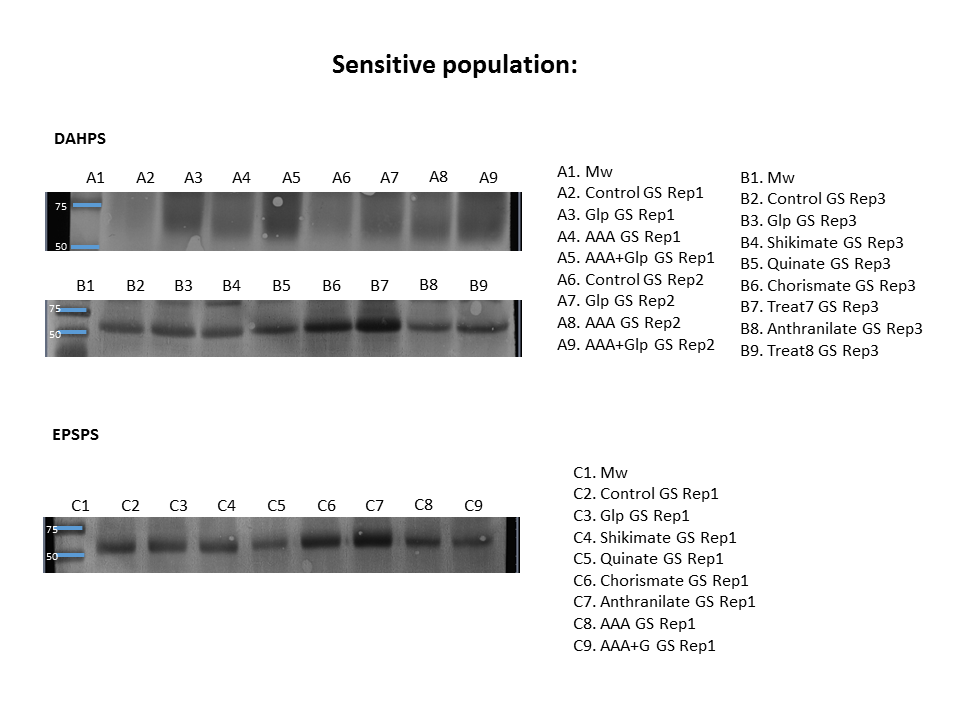
**

**Supplementary Figure 3:** Representative DAHPS and EPSPS immunoblots of glyphosate-sensitive Total soluble protein were fractioned by 12.5% SDS-PAGE and blotted.


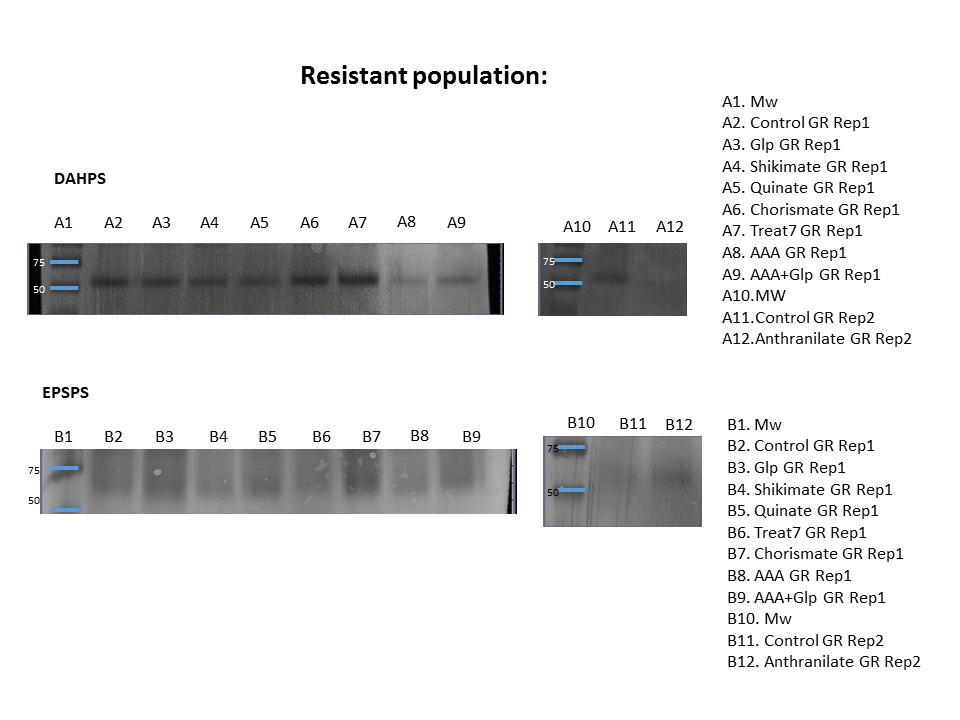


**Supplementary Figure 4:** Representative DAHPS and EPSPS immunoblots of glyphosate-sensitive Total soluble protein were fractioned by 12.5% SDS-PAGE and blotted.

**Supplementary Table 1.** Oligonucleotide sequences used for the qRT-PCR reactions. Genes of the shikimate pathway: D-arabinoheptulosonate 7-phosphate synthase (*DAHPS*), dehydroquinate synthase (*DHQS*), 3-dehydroquinate dehydratase/shikimate dehydrogenase (*DQSD*), shikimate kinase (*SK*), 5-enolpyruvylshikimate 3-phosphate synthase (*EPSPS*), chorismate synthase (*CS*), chorismate mutase (*CM*) and anthranilate synthase (*AS*). Normalization gene selected for this study was β tubulin. For each primer pair, the annealing temperature is given.

.

| GENE | FORWARD | REVERSE | ANNEALING TEMP |
| --- | --- | --- | --- |
| AAA biosynthetic pathway | | | |
| *DAHPS* | cctcataggatgataagggc | ctttgcatggcagcataacc | 55 |
| *DHQS* | gcattgttggctagggatcc | aacctcggccttgttttcac | 61 |
| *DQSD* | ggtgtactcaagcaaggagc | tgtggactcttactatggcc | 57 |
| *SK* | gattctgaagcacaaagcagc | cagttgttttcccagagccc | 55 |
| *EPSPS* | aatgctaaaggaggccttcc | tcaatctccacgtctccaag | 61 |
| *CS* | cttgatagaaggaggcctgg | gtttctttcctaggagtagtg | 61 |
| *AS* | tttggagggaaggttgtgcg | ctggtgagctttttccatgc | 52 |
| *CM1-3* | gaatccaagcccgcgtataa | cttcaatccaatcgtctcaacaag | 59 |
| *CM 2* | aagggtactgaagctgttcaag | tgtgctaatgaaggcggtaag | 59 |
| *ADHα* | accctcgctcttctctctatc | cggccgtgttggaattagta | 52 |
| *ADHβ* | cgggaatcttcctttcgtctc | aggttgagctgcgtcaatag | 59 |
| Normalization gene | | | |
| *βTUBULIN* | gatgccaagaacatgatgtg | tccacaaagtaggaagagttc | 61 |
|  |  |  |  |
